# Concurrent Incipient Fault Diagnosis in Three-Phase Induction Motors Using Discriminative Band Energy Analysis of AM-Demodulated Vibration Envelopes

**DOI:** 10.3390/s26010349

**Published:** 2026-01-05

**Authors:** Matheus Boldarini de Godoy, Guilherme Beraldi Lucas, Andre Luiz Andreoli

**Affiliations:** Department of Electrical Engineering, Sao Paulo State University (UNESP), Av. Eng. Luiz Edmundo Carrijo Coube, 14-01, Bauru 17033-360, SP, Brazilandre.andreoli@unesp.br (A.L.A.)

**Keywords:** low-cost sensors, three-phase induction motors, signal processing, AM demodulation

## Abstract

Three-phase induction motors (TIMs) are widely used in industrial applications, with bearings and rotors representing the most failure-prone components. Detecting incipient damage in these elements is particularly challenging. The associated signatures are weak and highly sensitive to variations, and their identification typically demands sophisticated filters, deep learning models, or high-cost sensors. In this context, the main goal of this work is to propose a new algorithm that reduces the dependence on such complex techniques while still enabling reliable detection of realistic faults using low-cost sensors. Therefore, the proposed Discriminative Band Energy Analysis (DBEA) algorithm operates on vibration signals acquired by low-cost accelerometers. The DBEA operates as a low-complexity filtering stage that is inherently robust to noise and variations in operating conditions, thereby enhancing discrimination among fault classes, without requiring neural networks or deep learning techniques. Moreover, the interaction of concurrent faults generates distinctive amplitude-modulated patterns in the vibration signal, making the AM demodulation-based algorithm particularly effective at separating overlapping fault signatures. The method was evaluated under a wide range of load and voltage conditions, demonstrating robustness to speed variations and measurement noise. The results show that the proposed DBEA framework enables non-invasive classification, making it suitable for implementation in compact and portable diagnostic systems.

## 1. Introduction

Among the electrical machines used in modern factories, three-phase induction motors (TIMs) stand out worldwide, serving as the primary driving force behind over 90% of this equipment. This widespread use is due to the following reasons: they are simple to build and have few parts compared to conventional combustion engines, they have a wide rotational speed and torque range, and they are durable and adaptable. These characteristics make TIMs versatile and reliable for use in a wide range of machines and equipment [1,2,3].

Also, two components in TIMs stand out as the most prone to failure: the bearings and the rotor. Bearings are not only among the most critical and widely used mechanical components—playing an irreplaceable role in rotating machinery—but also the primary contributors to system failures. Approximately 45–55% of electric motor failures result from bearing wear caused by metal-to-metal contact [4,5]. The rotor accounts for about 10% of equipment failures, typically due to breakage of the rotor bars or the end ring [6,7].

To enable early diagnosis of TIMs, thereby preventing unexpected failures in industrial production, numerous researchers have devoted significant effort over the years to developing non-invasive techniques for detecting damage in these devices [8,9,10]. However, in the initial stages of wear of critical components, conventional diagnostic methods often fail to identify faults. This limitation arises because incipient damage generates signals—acoustic, electrical, or vibrational—with very low amplitudes and impulsive or inconsistent behavior. Consequently, recent studies have focused on alternative approaches to detect and classify such early-stage failures, employing methodologies that integrate high-precision (and often higher-cost) sensors, multisensor data fusion, artificial intelligence, neural networks, and related techniques [11,12,13].

Current research methodologies present two main issues that warrant attention. The first concern relates to the procedures used to assess bearing and rotor wear during the data acquisition phase. In practice, many studies artificially induce faults by drilling holes in bearings or rotor bars, creating damage that differs substantially from conditions typically observed in industrial motors [14]. The second issue relates to the complexity of signal-processing techniques required to detect incipient faults. These approaches often rely on sophisticated filters or deep neural networks, demanding large datasets and extensive computational resources to overcome the inherent difficulty of identifying incipient damage-related signals [13]. Consequently, this research area would greatly benefit from a simpler, more reliable methodology capable of detecting incipient damage while simulating realistic wear conditions—precisely the focus of the present study.

The main objective of this work is to propose a novel, cost-effective approach for detecting incipient damage that avoids the complexities of artificial intelligence and deep learning-based models. To this end, a device was designed to induce controlled, early-stage wear in bearings, thus reproducing conditions closer to those encountered in industrial applications of TIMs. A distinct method from those commonly reported in the literature was also employed to generate rotor damage, using an abrasive disk to introduce slight surface wear and an incipient crack in a rotor bar. Two low-cost accelerometers mounted externally on the motor frame were used for vibration data acquisition, and a new demodulation-based signal-processing method was implemented.

Furthermore, unlike many previous studies that evaluate diagnostic methods only at a single nominal operating point, the proposed methodology was tested under multiple steady-state operating scenarios representative of industrial use. Three conditions were considered: full load (100% of the rated torque) with nominal balanced voltage, reduced load (80% of the rated torque) with nominal balanced voltage, and full load with a 5% supply-voltage unbalance. These scenarios naturally lead to different steady-state speeds due to slip variation with load and introduce realistic electrical and mechanical disturbances arising from the power source, DC generator loading, and mechanical couplings. In total, 2400 experiments were carried out combining these operating conditions with ten distinct health and fault configurations, providing evidence that the proposed DBEA features remain discriminative under practical variations in load and supply voltage.

This paper is organized as follows. Section 2 reviews previous works and relevant background. Section 3 discusses the application of AM demodulation to three-phase induction motor monitoring. Section 4 presents the DBEA-based fault diagnosis algorithm and its implementation. Section 5 describes the experimental setup and dataset acquisition procedures. Section 6 reports and discusses the experimental results. Finally, Section 7 summarizes the main conclusions and outlines directions for future work.

## 2. Previous Works

Although a wide variety of studies and methods have been proposed to non-invasively detect rotor and bearing failures [15], most do not employ components that have naturally worn through use, nor do they adopt failure detection methodologies that accurately reflect real operating conditions. In fact, the vast majority of these studies induce faults by drilling holes into the bearing’s outer race or the rotor bar [16,17,18,19,20,21,22]. While this approach is widely adopted in the scientific community, drilling artificially generates more severe and easily detectable faults than incipient damage. Therefore, it provides only a limited representation of the natural wear observed in industrial applications.

Other studies utilize prebuilt datasets to validate diagnostic methodologies, thereby avoiding the issue of artificially induced wear. One of the most widely used datasets is the rolling-bearing dataset provided by the Case Western Reserve University (CWRU) Bearing Data Center, which has been extensively employed in recent research [23,24,25,26]. However, the damage in this dataset was also artificially created: the motor bearings were damaged using electro-discharge machining (EDM), producing defects ranging from 0.007 in to 0.040 in in diameter, introduced separately at the inner raceway, rolling element, and outer raceway.

Recent deep learning-based approaches also rely heavily on these prebuilt datasets. For example, Pang et al. proposed an explainable and lightweight 1D convolutional neural network (ELCNN) designed to reduce the parameter count and computational complexity of conventional CNN architectures [27]. Although this yields a network that is relatively lightweight within the family of deep CNNs, it still requires supervised training and a non-negligible computational budget compared with simpler signal-processing-based techniques. Moreover, the experiments are conducted under essentially fixed operating conditions (constant speed and supply), without systematic variation in load or supply-voltage unbalance. On the other hand, the present work focuses on well-characterized incipient faults and explicitly considers different load and voltage scenarios in an experimentally acquired dataset.

A related deep learning-based approach was recently presented by Jiang et al., who proposed a dual-branch ConvNeXt network with an improved DenseBlock (DCN) for intelligent rolling-bearing fault diagnosis [28]. The DCN architecture is trained and evaluated on public datasets, including the CWRU rolling-bearing benchmark and an aero-engine intermediate bearing dataset, and its performance is compared against several deep models (ResNet, CapsNet, Inception, Transformer-based TST, ConvNeXt variants, etc.). Although the proposed network achieves high diagnostic accuracy, it still requires substantial computational resources (GPU-based training, CWT preprocessing, and a relatively deep convolutional architecture) and supervised learning on labeled datasets. Moreover, as in most CWRU-based studies, the bearing defects are introduced by EDM with fixed damage sizes (0.007–0.021 in) and the operating conditions correspond to a few discrete steady-state speeds under nominal supply, without systematic variation in mechanical load or supply-voltage unbalance, in contrast with the experimental protocol adopted in the present work, which focuses on realistically worn bearings, explicitly quantified incipient damage levels, and multiple load/voltage scenarios acquired on a dedicated test bench.

While Jalonen et al. [29] propose a CNN-based diagnostic scheme for seeded bearing faults under time-varying rotational speeds using the KAIST test rig dataset, their study focuses exclusively on bearing defects introduced in a controlled manner and evaluated under a limited set of operating conditions (variable speed, but without systematic variation in mechanical load or supply-voltage unbalance). Moreover, the approach relies on a deep learning architecture trained on high-quality laboratory accelerometer data, which requires supervised training and non-negligible computational resources. In the present work, we instead address naturally worn, strongly incipient faults occurring concurrently in both the bearings and the rotor of a three-phase induction motor, under multiple steady-state load levels and supply-voltage unbalance scenarios.

Unlike the previous works, the wear generated naturally in the bearings used in the present experiment—resulting from frictional contact among the bearing elements during operation—ranges from approximately 100 μm to 450 μm. Therefore, the faults examined in this study are over one hundred times smaller than those in the most commonly used dataset and were produced through a methodology that more closely replicates real-world operating conditions.

When examining studies that truly address incipient damage, it becomes evident that methodological complexity increases substantially. These works frequently employ advanced filters, neural networks, high-precision (and often costly) sensors, or composite diagnostic systems [30,31,32]. Such approaches, while effective in controlled environments, pose practical challenges for industrial implementation, as they demand systems that are compact, portable, versatile, and economically feasible—criteria that are difficult to meet simultaneously [33].

More recently, Lv et al. [34] proposed an incipient fault feature extraction method for rolling bearings based on signal reconstruction. Their approach combines the Teager–Kaiser energy operator with a Savitzky–Golay filter whose window length and polynomial order are optimized via a particle swarm optimization (PSO) algorithm, and the filtered TEO envelope is then multiplied by the original signal to obtain a feature-enhanced reconstruction suitable for subsequent sparse decomposition. Although this processing chain is effective at highlighting weak impacts under low SNR, it involves iterative global optimization and additional filtering and decomposition stages, leading to a substantially higher computational complexity than simple band energy features. From an experimental standpoint, the validation is carried out on a test rig using electrically machined defects (grooves of 1.2 mm depth and 0.6 mm width) on the inner and outer bearing races at a fixed motor speed of 600 rpm, without concurrent rotor faults, without naturally worn components, and without systematic variations of load or supply voltage.

In addition, Fort et al. [35] proposed a low-complexity rolling-bearing diagnosis technique tailored for embedded systems, combining time-domain preprocessing with a lightweight neural network classifier. In their approach, vibration and speed signals are transformed into fixed-size grayscale images, and these images are then classified by a small neural network. The method is trained mainly on emulated data and subsequently validated using the CWRU seeded-fault dataset, focusing on classical single-point defects in bearings (inner race, outer race, and rolling elements) without explicitly addressing strongly incipient damage or concurrent faults. Moreover, although the preprocessing is designed to reduce sensitivity to rotational speed and sampling frequency, no experimental results are reported with varying mechanical load or supply-voltage conditions. However, the present work concentrates on naturally worn, incipient bearing faults quantified via clearance measurements, concurrent rotor-bar and bearing faults, and an extensive set of 2400 experimental tests under different load levels and supply-voltage unbalance, all processed by a non-neural, band energy-based classifier.

Recent deep-learning-based works have also investigated fault detection under operating-condition variability. For instance, Spirto et al. [36] presented a comprehensive comparative study between SDP-CNN and time–frequency CNN strategies under different operating conditions, achieving strong diagnostic performance for bearing fault detection on the CWRU dataset. Importantly, the authors also reported computational time results, with the fastest evaluated configuration requiring 0.18 s, providing a useful reference for practical deployment considerations. Nevertheless, the study focuses only on bearing-related faults and does not address rotor-related conditions. In this context, while such CNN-based solutions are clearly effective, their overall processing chain is typically more computationally demanding than low-complexity signal-processing pipelines. The Results section shows that the proposed DBEA-based approach achieves substantially shorter execution times while also targeting fault concurrency, including broken-rotor-bar conditions, which are outside the scope of [36].

A second relevant example is the interpretable Wavelet Kolmogorov–Arnold Convolutional LSTM proposed by Chen et al. [37], which combines a wavelet-based time–frequency representation with a deep spatial–temporal feature extractor and an interpretable learning stage. Although this framework is efficient, it involves a multi-stage processing and learning pipeline. Moreover, the authors validated their method on three datasets: In the first one, the faults were incipient, yet rotor faults and fault concurrency were not considered. In the second one, despite speed variation, the bearing was not installed in the motor itself but in a gearbox-type rotating system (with no rotor-related faults). Finally, in the third dataset, the faults were visually evident in the pictures due to their severity, again without rotor faults. These aspects reinforce the relevance of studying concurrent incipient bearing wear and broken-rotor-bar faults under realistic operating conditions using a computationally feasible pipeline, which is the central focus of the present work.

The method proposed in this research is based on the Fast Fourier Transform (FFT), AM demodulation, and a lightweight search (DBEA) to obtain a discriminative yet low-complexity diagnostic pipeline from raw vibration measurements. First, the vibration signal is demodulated to extract its envelope, which emphasizes impulsive and modulation-related components typically associated with early-stage mechanical degradation and makes fault signatures more evident than in the raw waveform. Next, the envelope is mapped to the frequency domain via the FFT, yielding a compact representation where fault-related energy concentrates around characteristic bands and sidebands.

Rather than relying on manually selected frequency bins or computationally intensive feature-learning models, the proposed DBEA stage automatically searches for the most informative frequency regions and defines a small set of discriminative band energy features. This search-driven band selection increases robustness to operating-condition variability, such as different load levels and supply-voltage unbalance. The features are built from energy aggregated over bands, which are typically less sensitive to slight spectral shifts and measurement noise than single-line indicators. Finally, these few band energy features feed a simple classifier, resulting in a method that is both interpretable and suitable for real-time deployment due to its reliance on efficient signal-processing operations and a compact feature set. Overall, this framework offers an attractive trade-off between practical feasibility and diagnostic performance.

The limitations of current research—namely the use of large, non-incipient faults and overly complex methodologies—apply to both bearing and rotor failure detection. Therefore, this study seeks to address both types of incipient failures simultaneously. Thus, the method was also designed to prevent one fault type from masking the other and to enable their concurrent detection.

The main scientific contributions of this study are as follows:

- Detection of incipient faults non-invasively, even under varying operating conditions: the proposed method enables early identification of internal damage in TIMs using external vibration measurements, without the need to stop the equipment or disassemble the motor. This facilitates advanced maintenance scheduling and helps prevent unexpected breakdowns that halt production and cause financial losses, even in the presence of load and supply-voltage variations that are known to affect accelerometer-based diagnostics [38,39].
- Low-cost sensors: Increased economic viability of applying the method in practice and manufacturing dedicated equipment.
- A new approach through signal demodulation requires less processing power, allowing for implementation on portable devices and offering greater flexibility.
- Component wear is similar to natural wear: the failures are incipient and much closer to what is found in real applications than most failures reported in the literature.

## 3. AM Demodulation Applied to Three-Phase Induction Motor Monitoring

Amplitude modulation (AM) is widely used in radio communications. However, faults in three-phase induction motors can also modulate electrical and mechanical variables (such as current, sound, vibration, and magnetic fields) in a manner analogous to communication signals [40]. Fault-related frequencies may modulate not only the fundamental components (e.g., the 60 Hz supply frequency in the current) but also each other. For instance, a fault that induces high-frequency vibrations can mechanically modulate the motion associated with a fault that produces lower-frequency vibrations. As a result, the vibration signals acquired by accelerometers become amplitude-modulated by the combined effect of these fault mechanisms.

In this framework, since rotor and bearing faults invariably induce distinct characteristic fault frequencies, the upper and lower envelopes of the vibration signals also differ for each fault type. At the same time, the proposed AM-based fault detection method is largely insensitive to motor loading because speed variations shift the fault-related frequency components of both fault types in a similar manner. Similarly, electrical and mechanical noise sources follow the same logic: by affecting the fault frequencies of both fault types in an approximately equal way, they have little impact on the classification. Therefore, the proposed AM-based modulation method effectively acts as a noise filter and does not rely on additional complex filtering structures, which is one of the key reasons it remains sensitive to highly incipient faults in both the bearings and the rotor, as shown in the results.

### Analytical Effect of Speed Variations on Fault Frequencies

To illustrate the previous discussion, two types of faults were modeled: an inner-race bearing fault and a broken-rotor fault. The inner-race bearing fault frequency (BPFI) can be written as a linear function of the shaft rotational frequency $f_{r}$ (in Hz) [41].

(1) $$f_{\mathrm{BPFI}}\left(f_{r}\right)=\frac{n}{2}\left(1+\frac{d}{D}\cos\phi \right)f_{r}=K_{B}\;f_{r},$$

where *n* is the number of rolling elements, *d* is the rolling element diameter, *D* is the pitch diameter, ϕ is the contact angle, and $K_{B}$ is a constant that depends only on the bearing geometry.

Let $f_{r0}$ be a nominal rotational frequency. In induction motors, speed variations due to load changes typically result in a reduction in the mechanical speed. This effect can be modeled as

(2) $$f_{r}=L_{S}f_{r0},$$

where $L_{S}$ represents a small speed decrease index ($0<L_{S}<1$).

Substituting into (1), the BPFI under speed variation becomes

(3) $$f_{\mathrm{BPFI}}=K_{B}L_{S}\;f_{r0}.$$

Thus, a decrease in speed produces the same relative decrease in the inner-race bearing fault frequency.

On the other hand, broken-rotor-bar (BRB) faults can generate sideband components around the electrical supply frequency $f_{e}$ in vibration/envelope spectra [42]:

(4) $$f_{\mathrm{BRB},\pm }=f_{e}\left(1\pm 2s\right),$$

where $f_{e}$ is the electrical supply frequency (Hz) and *s* is the slip. Let *p* denote the number of pole pairs (p=P/2). The mechanical synchronous rotational frequency is

(5) $$f_{\mathrm{syn}}=\frac{f_{e}}{p},$$

and the slip can be written in terms of the mechanical speed $f_{r}$ (Hz) as

(6) $$s=\frac{f_{\mathrm{syn}}-f_{r}}{f_{\mathrm{syn}}}.$$

substituting (5) and (6) into (4), the BRB sidebands expected in the measured spectrum become

(7) $$f_{\mathrm{BRB},+}=3f_{e}-2pf_{r},$$

(8) $$f_{\mathrm{BRB},-}=-f_{e}+2pf_{r}$$

Thus, the BRB components appear as affine functions of $f_{r}$ and they form symmetric sidebands around $f_{e}$ whose spacing is $2sf_{e}$.

Also, from the previous equations, both the inner-race bearing fault frequency and the BRB-related sideband frequencies can be written as affine functions of the mechanical speed $f_{r}$. Consequently, a change in speed shifts the fault-related spectral components approximately linearly with $\Delta f_{r}$. When using amplitude-modulated envelope spectra and relatively wide frequency bands, this common linear shift tends to affect different fault components in a similar way. As a result, the relative band energy distribution remains approximately invariant, and the fault-related components stay within the width of the analyzed frequency bands. This supports the assumption that the proposed AM-based band energy classification is weakly sensitive to speed (or load) variations.

The analytical relations above show that the characteristic frequencies associated with both bearing and rotor faults vary approximately affinely with the mechanical speed $f_{r}$, and therefore with the load level. For the 6205 bearing (SKF, Cajamar, Sao Paulo, Brazil), the inner-race fault frequency satisfies $f_{\mathrm{BPFI}}\left(f_{r}\right)=K_{B}f_{r}$ with $K_{B}\approx 5.41$, so a moderate load-induced speed decrease from 1740 rpm to 1720 rpm shifts $f_{\mathrm{BPFI}}$ from approximately 157.0 Hz to 155.2 Hz. For broken-rotor-bar (BRB) faults, the low-frequency modulation component in the envelope spectrum can be written as $f_{m}=2sf_{e}$. Under the same speed change, $f_{m}$ shifts from about 4.0 Hz to 5.3 Hz. Hence, the relevant fault-related components drift with operating point, but the drift is well described by affine (approximately linear) relations with respect to $f_{r}$.

Thus, in the envelope spectrum, spectral components at $f_{\mathrm{BPFI}}$ (and their harmonics $2f_{\mathrm{BPFI}},3f_{\mathrm{BPFI}},\ldots$), the BRB-related modulation component $f_{m}=2sf_{e}$, and cross-terms produced by amplitude modulation such as $f=f_{\mathrm{BPFI}}\pm f_{m}$ and, more generally, $f=kf_{\mathrm{BPFI}}\pm f_{m}$ with k=2,3,… should be expected (for each operating speed). Although these components appear at different absolute frequencies as the load changes, their locations remain governed by the same affine dependencies on $f_{r}$. This observation motivates a band-based representation. By aggregating envelope-spectrum energy over frequency bands, it becomes possible to identify bands that remain discriminative across a wide range of load levels. Importantly, even though the characteristic fault frequencies can be computed analytically, the strongly incipient nature of the investigated defects makes diagnosis based solely on a simple FFT peak-search (or narrowband inspection) unreliable in practice, because the associated spectral components may exhibit very low amplitudes and energy spreading across neighboring bins due to noise. This limitation is later corroborated in the manuscript by the quantitative comparison against conventional low-complexity indicators and envelope-spectrum features, which show substantially lower discriminative performance than the proposed approach.

Therefore, the most significant bands can be selected by measuring which bands exhibit the largest systematic discrepancies among fault classes across experiments, even when $f_{r}$ varies between operating points. This principle underlies the proposed Discriminative Band Energy Analysis (DBEA) approach and is detailed in the next section.

## 4. DBEA-Based Fault Diagnosis: Algorithm and Implementation

Based on the behavior described in Section 3, an algorithm was developed to detect and classify faults under all operating conditions.

In this framework, the upper and lower envelopes were obtained in MATLAB^®^ (2025a), and their spectral content was computed for each of the 2400 experiments. Subsequently, an algorithm termed Discriminative Band Energy Analysis (DBEA) was implemented to calculate the energy in predefined frequency bands of these envelope spectra and to classify the faults based on band energy. In this sense, the algorithm identifies, for each condition, the bands that exhibit the largest energy variations, thereby maximizing the discrimination capability between fault classes. In addition, several algorithm parameters were investigated, including the frequency bands, the envelope window length, and the envelope computation method.

For instance, let e(t) denote the vibration envelope signal, previously computed in MATLAB^®^ by any suitable demodulation technique (e.g., Hilbert-based or otherwise). In discrete time, an envelope segment of duration $T_{\mathrm{env}}$ is represented by

(9) e[n],n=0,1,…,N−1,

with a sampling frequency of $f_{s}$ and

(10) $$T_{\mathrm{env}}=\frac{N}{f_{s}}.$$

A window function w[n] is applied to the envelope to control spectral leakage, and the *N*-point discrete Fourier transform (DFT) of the windowed envelope is computed as

(11) $$E\left[k\right]=\sum_{n=0}^{N-1}e\left[n\right]\;w\left[n\right]\;e^{-j2\pi kn/N},\;k=0,1,\ldots ,N-1.$$

The corresponding frequency resolution is

(12) $$\Delta f=\frac{f_{s}}{N}=\frac{1}{T_{\mathrm{env}}},$$

and the frequency associated with bin *k* is

(13) $$f_{k}=k\;\Delta f.$$

The DBEA algorithm partitions the envelope spectrum into contiguous frequency bands of width *B* (the analysis bandwidth). For uniformly spaced bands, the *b*-th band is defined by

(14) $$f_{b}^{L}=\left(b-1\right)B,\;f_{b}^{H}=bB,$$

and the corresponding set of spectral indices is

(15) $$J_{b}=\left\{k\;|\;f_{b}^{L}\leq f_{k}<f_{b}^{H}\right\}.$$

The band energy for band *b* is then computed as

(16) $$W_{b}=\sum_{k\in J_{b}}{\left|E[k]\right|}^{2}\;\Delta f,$$

where the factor Δf accounts for the frequency bin width. Since $\Delta f=1/T_{\mathrm{env}}$, the approximate number of frequency bins contributing to each band is

(17) $$\left|J_{b}\right|\approx \frac{B}{\Delta f}=B\;T_{\mathrm{env}},$$

explicitly linking the band energy $W_{b}$ to both the bandwidth *B* and the envelope window length $T_{\mathrm{env}}$.

Using the final parameter configuration ($B,T_{\mathrm{env}}$), the resulting band energy feature vector $W={\left[W_{1},\;W_{2},\;\ldots ,\;W_{N_{\mathrm{bands}}}\right]}^{T}$ was used to construct classification feature-space maps by mapping the three most significant band energy components onto the x-, y-, and z-axes, enabling diagnosis even in incipient fault scenarios.

**Figure 1:**
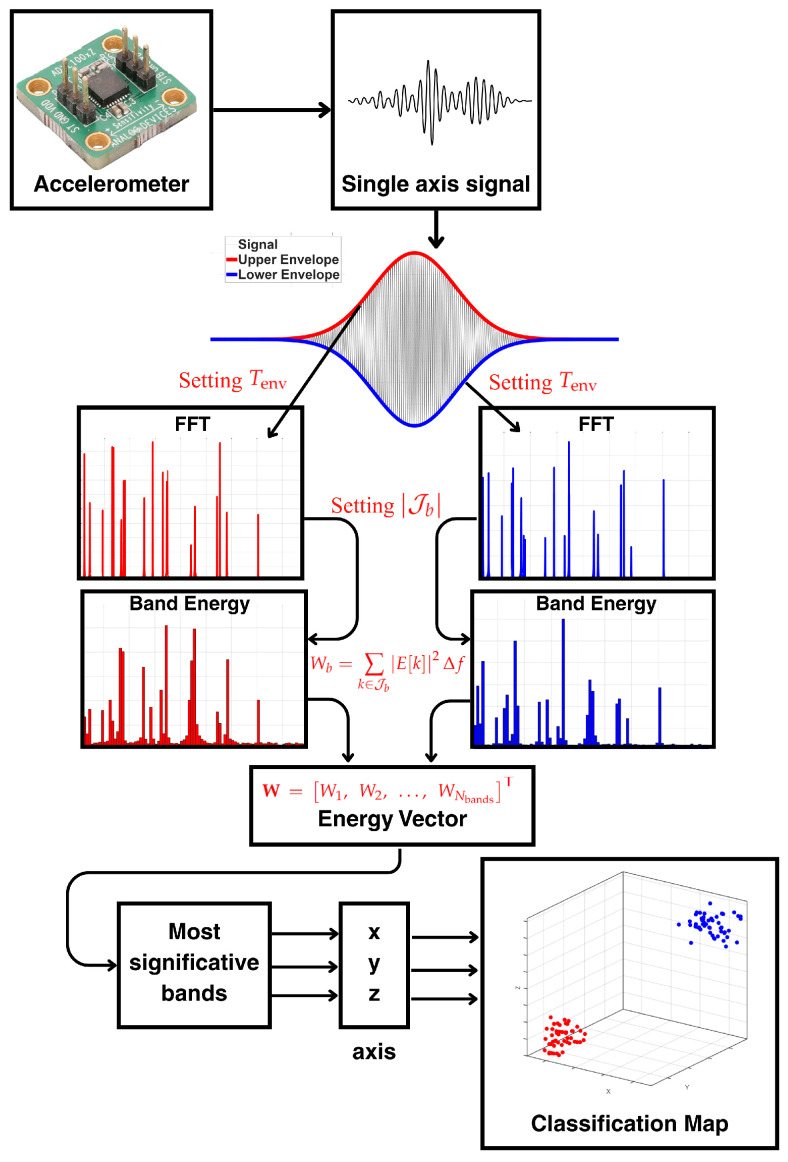
Algorithm flowchart.

Finally, the diagram in Figure 1 illustrates the steps involved in signal processing and classification. First, a single-axis accelerometer was used to acquire the vibration data during the experiments. Next, the envelope window length $T_{\mathrm{env}}$ was selected based on the physical model described in Section 3, ensuring a time interval longer than the characteristic fault periods for the tested motor-bearing combination. Then, the band energy vector *W* was computed after defining the band index $J_{b}$ (the parameter selection was discussed in Section 6). Finally, the most significant bands were identified by selecting those $W_{b}$ values that exhibited the largest discrepancies among the different operating conditions. Two single-axis accelerometers were used in the experiments, yielding two vibration channels in total. One sensor was mounted and oriented to measure radial acceleration on the motor housing, while the other was oriented to capture transverse acceleration, as shown in Figure 1. The proposed signal-processing pipeline is applied independently to each channel, so that the diagnostic procedure can be carried out using either the radial or the transverse vibration signal.

**Figure 2:**
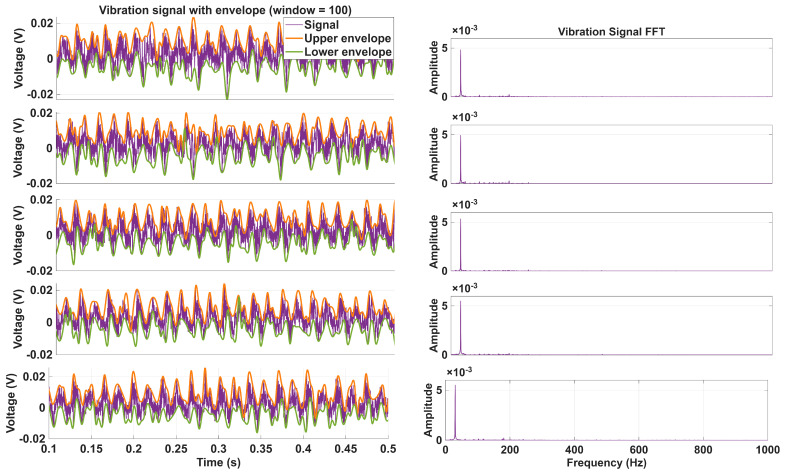
Vibration signals (**left**) and their corresponding FFT magnitude spectra (**right**) for five different conditions.

In Figure 2, five samples of real acquired vibration signals are shown together with their upper and lower envelopes on the left, and the FFT magnitude spectra of those same signals are presented on the right. It can be observed that, in a real test bench environment, the measured vibration signals contain background noise and incidental disturbances, and that the fault-related spectral components are much smaller than the dominant mechanical vibration component because the investigated faults are strongly incipient.

## 5. Experimental Setup and Dataset Acquisition

This section describes the methodology adopted, including the procedures used to introduce wear in the bearing and rotor, the experimental setup, and the implementation of the algorithm.

### 5.1. Component Wearing

**Figure 3:**
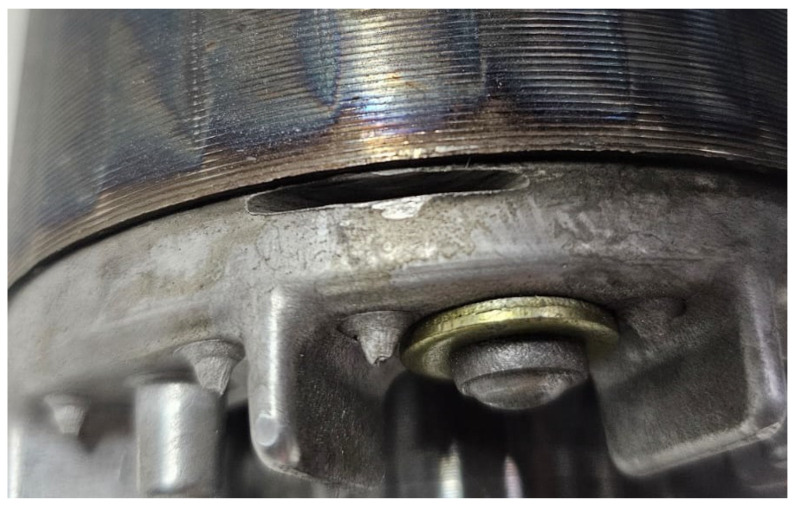
Close view of the damaged rotor.

Figure 3 shows the minor damage introduced in the TIM rotor. An abrasive rotary disk was used to wear the tip of the rotor ring until it touched the bar, producing a small crack. In this way, an incipient failure can be emulated, and the proposed fault detection method can be evaluated.

**Figure 4:**
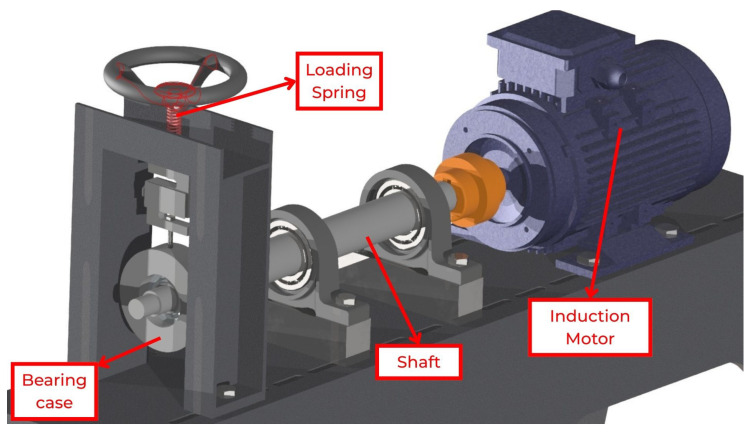
Bearing wear machine to simulate natural wear.

To produce bearing wear, a dedicated test rig was developed, as shown in Figure 4. It consists of a metallic base, a motor, a shaft, and a loading spring acting on the bearing. For each bearing, one side of the shield was removed, and the interior was cleaned with a solvent to remove all grease. A fine abrasive paste was then applied in place of the grease, and the bearing was mounted on the shaft driven by the motor. By turning the crank, a radial load was applied to the bearing, thereby simulating realistic operating loads. The bearings were regreased before the data collection tests.

**Figure 5:**
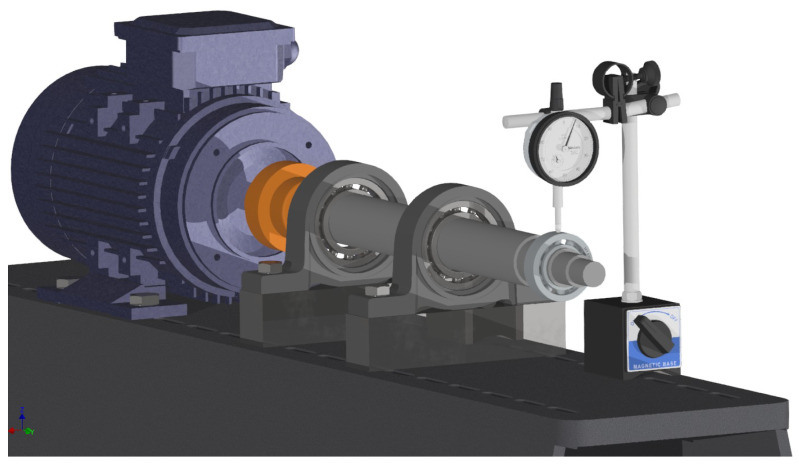
Dial indicator for measuring bearing wear.

Four different bearings were worn, and their internal clearance (which is proportional to the wear level) was measured using a precision dial indicator (Figure 5). The instrument has a resolution of 10 μm and was rigidly fixed to the metallic base of the test rig, while each bearing under test was mounted on the shaft as shown in Figure 5. All measurements were performed with the bearings at room temperature. For each bearing, the shaft was slowly rotated while continuously monitoring the dial indicator, and any reading exceeding the manufacturer’s tolerance was taken as evidence of localized damage. The clearances obtained, in ascending order, were as follows: 100–130 μm, 150–180 μm, 200–250 μm, and 450–480 μm. Because these values are very small, ten independent measurements were taken for each bearing, and the reported ranges correspond to the minimum and maximum readings observed across these ten repetitions.

To relate these values to the incipient nature of the faults, all bearings were inspected both before and after the wear process. Prior to wear, none of the bearings exhibited variations exceeding the manufacturer’s tolerance of 10 μm, indicating that they were within normal operating clearances. After wear, all bearings presented peak clearances above this tolerance, with different severity levels corresponding to the ranges reported above. In this way, the depth of the induced damage is quantified directly in terms of the increase in internal clearance beyond the nominal tolerance, confirming that the faults investigated in this study are incipient but clearly distinguishable from healthy bearings.

A brand-new bearing and a brand-new rotor were also used in the experiments as reference conditions, enabling fault separation and the analysis of possible masking effects between rotor and bearing faults.

### 5.2. Machinery Setup

**Figure 6:**
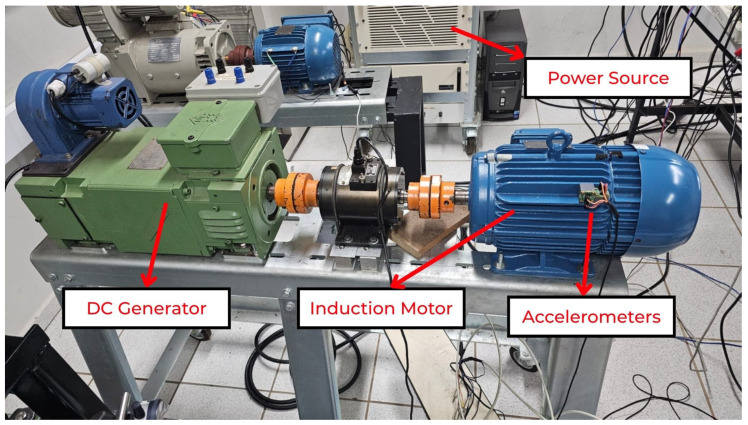
Electrical machines picture.

For the tests, a DC machine and a TIM were employed. The DC machine field winding was supplied by a variable-voltage autotransformer (VARIAC) followed by a bridge rectifier, and the armature terminals were connected to a resistor bank. In this way, by varying the field current, the effective loading could be precisely controlled. The load values were monitored using electrical meters. The TIM was powered by a 360 AMX programmable power source (Pacific Power, Irvine, CA, USA). This device allows the voltage of each phase, as well as the waveform and phase shift between phases, to be independently adjusted, thus enabling controlled voltage drop conditions to be tested. Figure 6 shows the coupling between the machines.

Table 1 summarizes the rated parameters of the induction motor and the DC generator used in the experimental test bench, including the bearing type installed on the motor shaft.

In all experiments, the induction motor was fitted with a single-row deep-groove ball bearing SKF 6205 at the drive end. This bearing has a nominal bore diameter of 25 mm, an outer diameter of 52 mm, and a width of 15 mm.

Using the programmable power source, the phase voltages could be adjusted independently, while the mechanical load was set via the VARIAC. For each combination of the five bearings (one new and four progressively worn) and the two rotors (one damaged and one healthy), three operating scenarios were evaluated: full load with nominal voltage, reduced load with nominal voltage, and full load with a 5% voltage unbalance, as summarized in Table 2. Each operating condition was repeated 80 times, as indicated in the Repetitions column of Table 2, resulting in a total of 2400 vibration records.

These values were selected to approximate typical motor operating conditions in which it is very common for machines to operate between 80% and 100% of their rated load. A voltage imbalance of up to 5% is also frequently observed in industrial environments and often occurs during normal operation.

### 5.3. Sensors and Signal Acquiring

Two microelectromechanical system (MEMS) accelerometers EVAL-ADXL1005Z (Analog Devices Inc., Shanghai, China)were selected. Each device is a single in-plane axis accelerometer with an analog output, featuring a full-scale range of ±100 g and a linear frequency response from 0 to 23 kHz. MEMS accelerometers exhibit inherent noise, i.e., random fluctuations in the output signal, even when the input is constant. This noise is an intrinsic property of MEMS devices, arising from various physical phenomena and limiting their sensitivity and accuracy. Therefore, the MEMS used in this experiment were chosen for their ultra-low noise density of 75 µg/*√*Hz. This sensor can be considered low-cost, as it is commercially available at a price below USD50.00, in contrast to other vibration sensors that can exceed USD1000.00.

**Figure 7:**
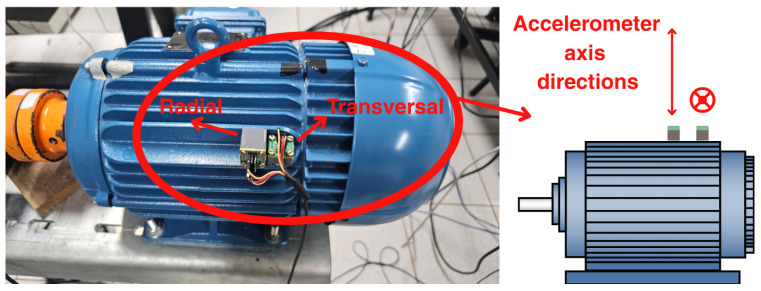
Accelerometers sensors on the TIM.

In all tests, the vibration signal was acquired using a single-axis accelerometer mounted on the external motor housing by means of a small magnetic base, as shown in Figure 7, with the sensitive axis of one sensor aligned in the radial direction and that of the other aligned in the tangential direction. This mounting technique is widely adopted in vibration-based condition monitoring of rotating machinery. Given the relatively small magnet and its location on the outer frame, away from the main stator–rotor flux path, its influence on the internal air-gap flux is expected to be negligible compared with the fundamental excitation and the small perturbations introduced by incipient faults. Anti-aliasing filters were employed together with shielded cables to ensure signal integrity.

**Figure 8:**
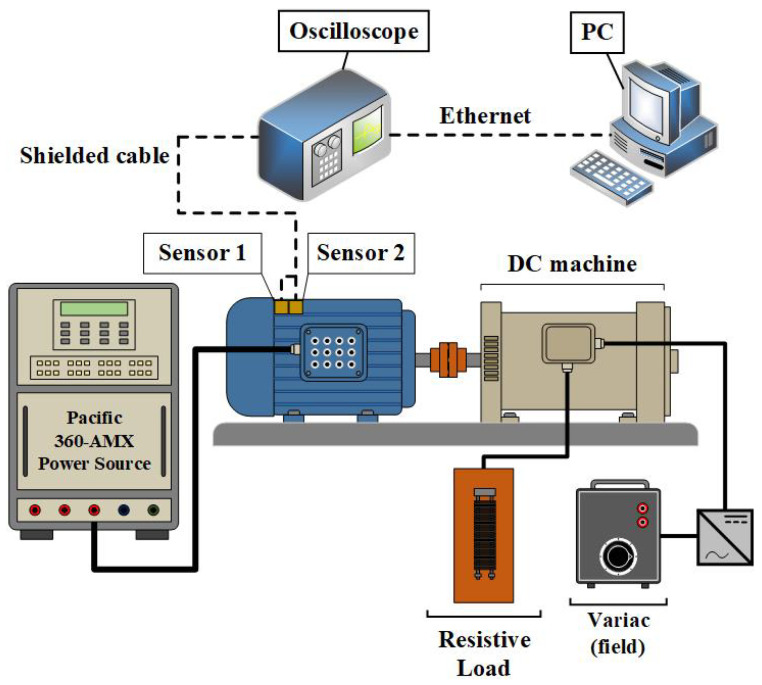
Experiment setup.

With the complete setup shown in Figure 8, a 850DL oscilloscope (Yokogawa, Japan) was used to acquire the signals from both accelerometers for each experimental condition, resulting in 2400 experiments. During acquisition, a sampling frequency of 500,000 samples/s was adopted to ensure oversampling and to avoid aliasing of any spectral components below 40 kHz. However, for the purposes of feature extraction and classification, the signals were numerically low-pass filtered and downsampled so that the effective analysis bandwidth was limited to 40 kHz, which is compatible with typical industrial vibration monitoring systems and low-cost data acquisition hardware. From a multirate signal-processing standpoint, a properly filtered and decimated sequence is equivalent, in the band of interest, to directly sampling the original bandlimited signal at the lower rate [43,44]. Thus, the proposed DBEA pipeline operates on data that could have been acquired directly at 40 kHz. The processed data were transferred to a personal computer for signal analysis using MATLAB^®^ software.

## 6. Results and Discussion

As discussed in the previous sections, the DBEA index can be used to classify incipient faults in the bearings and rotors of three-phase induction motors. In the present study, the most relevant results were obtained with a bandwidth of B=50 Hz and an envelope window length of 100 samples. To further support this choice, Table 3 reports a parametric analysis for a set of representative combinations, using the normalized average DBEA value as a performance indicator. As shown in Table 3, the configuration B=50 Hz and 100 samples provides the highest overall performance among the tested cases, while the remaining combinations preserve the same general clustering behavior with reduced average discrimination.

It is important to emphasize that, in the feature-space maps, the points associated with each condition (plotted in the same color) were acquired under different operating conditions, as described in Section 5. Despite variations in load, speed, and torque, these points remained consistently clustered because they correspond to the same fault type. This empirical behavior corroborates the theoretical robustness of the proposed DBEA-based features to noise and operational variations, as discussed in Section 3. Furthermore, it should be noted that the incipient fault depths investigated in this study are considerably smaller than those typically employed in most previous works, as discussed in Section 2.

**Figure 9:**
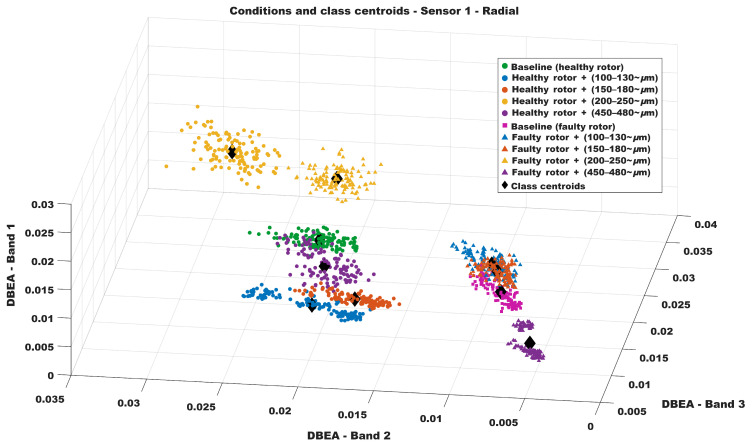
Classifying Map—Sensor 1.

Figure 9 shows the resulting three-dimensional classification feature-space map obtained from the DBEA values for sensor 1. The three axes represent the three most discriminative band energy features automatically selected by the proposed algorithm. Overall, a clustering behavior can be observed for samples associated with the same fault condition, which supports the subsequent definition of class centroids used for validation.

However, this 3D visualization should be regarded as a strong qualitative indication that the proposed method is capable of diagnosing concurrent incipient faults, rather than as absolute evidence of class separability by itself. Therefore, the most relevant evidence of diagnostic effectiveness is provided by the quantitative evaluation (randomized testing and multiple performance metrics), as subsequently reported in the confusion matrices and the corresponding class-wise scores.

A few groups may lie in close proximity in the feature-space maps. This behavior is expected in the present study because some investigated incipient bearing wear levels differ by only a few tens of micrometers. This effect is consistent with the residual confusions observed mainly between neighboring incipient classes, as will be shown in the subsequent sections.

**Figure 10:**
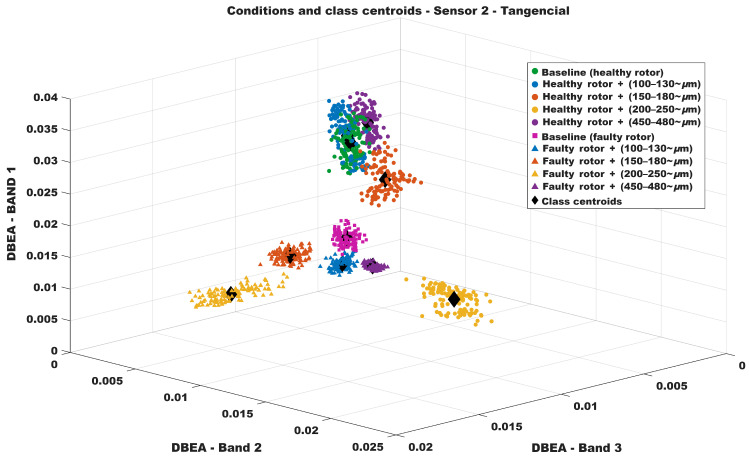
Classifying Map—Sensor 2.

Figure 10 shows the resulting three-dimensional classification feature-space map obtained from the DBEA values for sensor 2. As in the previous case, the three axes of the plot represent the three most significant bands according to the developed algorithm. The same clustering behavior can be observed among the DBEA values associated with each fault condition, allowing the corresponding centroids to be defined and subsequently used for validation. It is worth noting that, even though sensor 2 measures vibration along a different axis of the motor, the proposed technique remains valid since the theoretical principles discussed in Section 3 apply equally to any vibration direction.

**Figure 11:**
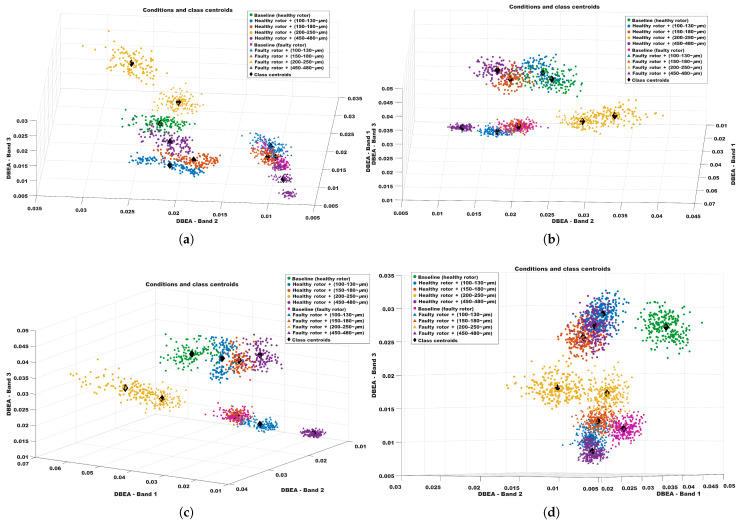
Three-dimensional DBEA feature space for the ten operating conditions using different cluster combinations. (**a**) Result for different bandwidths. (**b**) Result for different envelope windows. (**c**) Result for different envelope windows. (**d**) Result for experiment with smaller sampling time (more points).

As previously mentioned, the parameters can be adjusted within a reasonable range without significantly affecting the results. The following maps (Figure 11) show the outcomes of varying the envelope window, the bandwidth, and even the sampling time. By examining these results, the clustering behavior is clearly evident, indicating that, as long as the bandwidth is not smaller than the variation in the fault frequencies caused by changes in operating conditions, the DBEA algorithm will successfully classify the faults.

### 6.1. Testing

To validate the DBEA-based classification method, a set of randomly selected samples containing vibration signals for all fault types was used (20% of the full dataset). For each selected sample, the envelope was computed using the final parameter configuration, and the DBEA features were extracted from the three most significant bands. These three values form a point in a three-dimensional feature space.

For classification, the class centroids previously shown in the clustering maps were computed from the remaining samples (using the other 80% of the dataset). Then, for each test sample, the Euclidean distance from its 3D feature point to all centroids was calculated, and the sample was assigned to the class associated with the nearest centroid (nearest-centroid classifier).

**Figure 12:**
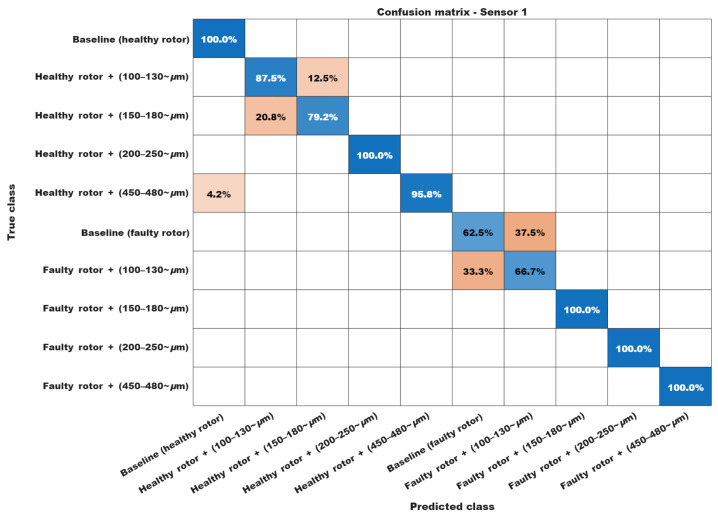
Confusion chart for Sensor 1.

**Figure 13:**
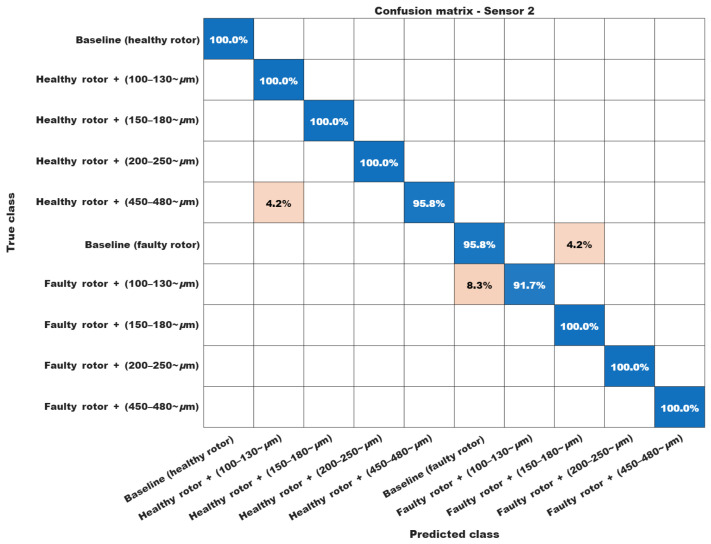
Confusion chart for Sensor 2.

Finally, the confusion matrices in Figure 12 and Figure 13 were obtained by comparing the predicted labels with the corresponding ground-truth labels for the test subset, and are reported as percentage values for readability.

Some classification percentages merit further comment. First, the algorithm exhibits a certain degree of confusion between the 100–130 μm and 150–180 μm bearing fault conditions. This behavior is, to some extent, expected, since a 20 μm difference between these conditions is very small, considering that even 480 μm still corresponds to an incipient damage level. A similar behavior is observed in the confusion between the healthy bearing and the 100–130 μm bearing fault when the rotor is damaged.

Finally, the only deviation that may be regarded as a misclassification is the 4.2% confusion between the 450–480 μm condition and the healthy motor condition. However, this rate is still quite acceptable from a practical application standpoint.

For sensor 2, the confusion matrix in Figure 13 exhibits slightly better performance than that obtained for sensor 1. Nonetheless, some misclassification still occurs between closely spaced fault levels. As in the previous analysis, however, the corresponding percentages remain fully compatible with those reported in related works and are acceptable for practical applications. These results further confirm that the DBEA technique is robust and can be regarded as a viable alternative for detecting strongly incipient faults in three-phase induction motors.

### 6.2. Performance Comparison

**Figure 14:**
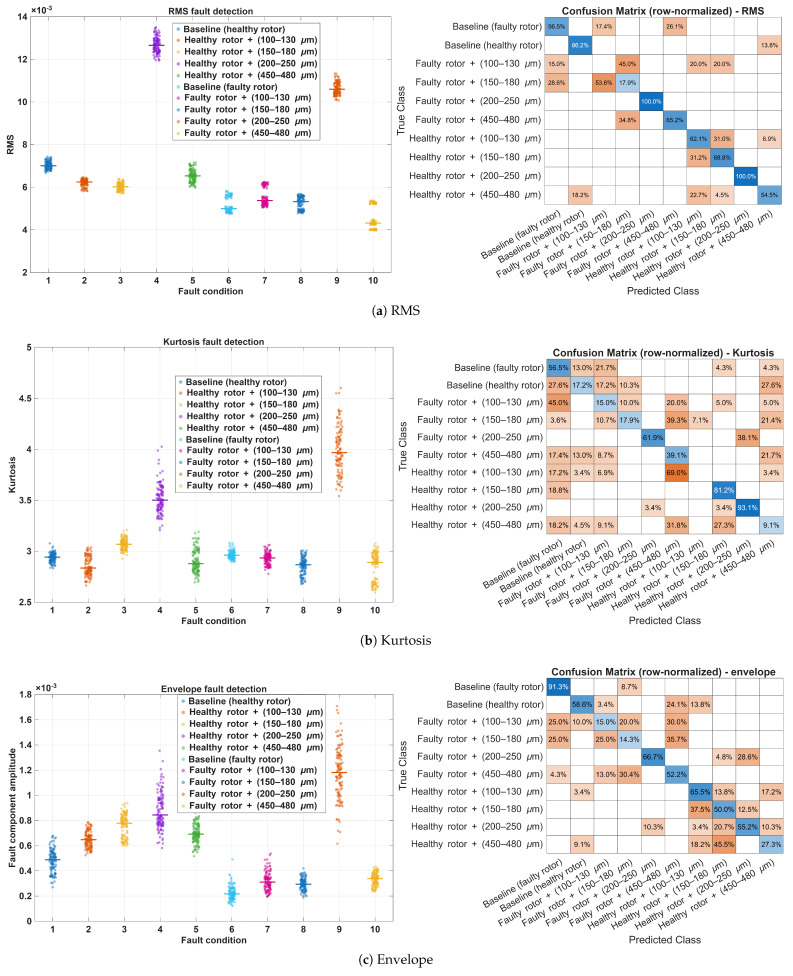
Analysis of evaluated methods.

To further assess the performance of the proposed DBEA-based features, three commonly used vibration indicators were also evaluated as baseline methods: root-mean-square (RMS) values, kurtosis, and conventional envelope-spectrum band energies. For each approach, features were extracted from the same vibration signals adopted in the DBEA analysis, and a simple centroid-based classifier was applied to ensure a fair comparison among all methods. Figure 14 illustrates the resulting feature spaces obtained from (a) RMS-based features, (b) kurtosis-based features, and (c) envelope-analysis features, using the same representation adopted for the DBEA feature-space maps. In all three cases, the points associated with different operating conditions exhibit strong overlap, especially when comparing closely spaced incipient bearing faults and combinations of bearing and rotor damage. In contrast, the DBEA feature space shows well-defined clusters for the same ten operating conditions.

Also, Table 4 reports the class-wise accuracy (Acc), precision (P), recall (R), and F1-score for the proposed DBEA-based method and for the three baseline approaches, expressed as mean ± standard deviation over the 10-fold evaluation. Since the class-wise accuracy is computed in a one-vs.-rest manner, it may remain high due to the large number of true negatives in the multi-class setting. Therefore, per-class precision/recall/F1 are more informative for analyzing class confusions. Overall, the baseline methods exhibit limited discriminative capability for the considered scenario, with macro-averaged F1-scores of 0.60 (RMS), 0.37 (kurtosis), and 0.45 (envelope-analysis), and overall accuracies of 0.61±0.032, 0.40±0.028, and 0.47±0.042, respectively. In contrast, the DBEA-based method reaches an overall accuracy of 0.93±0.024 and a macro-averaged F1-score of 0.93, while also presenting high class-wise accuracies for most conditions (typically ≥0.95), with several classes achieving perfect scores across folds (Classes 4 and 8–10). The remaining performance degradation is concentrated in a small subset of closely spaced incipient fault levels (notably Classes 6–7), for which the class-wise accuracies remain high (0.95±0.020) but the precision/recall/F1 values decrease, reflecting the increased confusion between neighboring incipient conditions (100 μm).

Overall, these results indicate that conventional scalar indicators are insufficiently discriminative for strongly incipient concurrent faults, whereas the proposed DBEA band energy features provide a more informative representation for classification.

**Figure 15:**
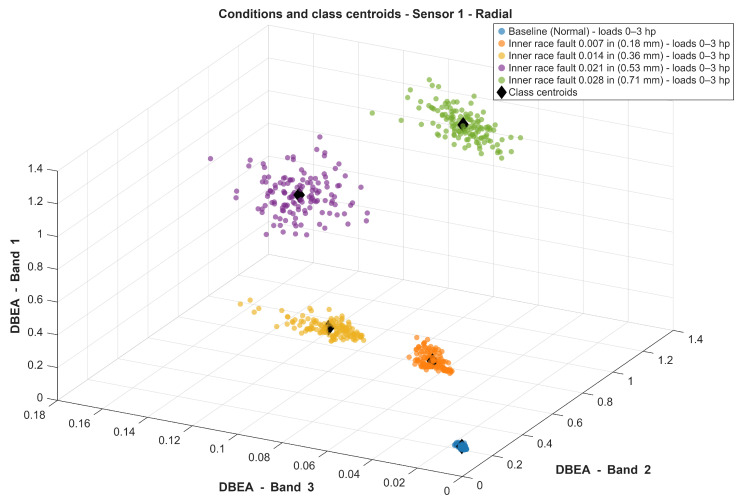
Three-dimensional Map for external dataset.

In addition, the same FFT+AM+DBEA pipeline was applied to the public CWRU bearing fault dataset under five representative operating conditions. The corresponding three-dimensional DBEA feature-space map (Figure 15) shows a clear separation trend among the five classes, while the quantitative evaluation using repeated stratified 10-fold cross-validation (10 × 10) is summarized in Table 5. Overall, the method achieves 0.97±0.020 accuracy, with strong class-wise performance (average precision/recall/F1 of 0.97/0.97/0.97). Most classes exhibit near-perfect indices, whereas the remaining reductions are mainly associated with one class (Class 2), for which the recall is lower, indicating a limited amount of confusion with neighboring conditions. This complementary experiment indicates that the proposed DBEA framework is not only capable of distinguishing strongly incipient, naturally worn faults in the experimental test bench, but also performs even better when applied to larger faults such as those present in the CWRU dataset, thereby reinforcing both its robustness and its general applicability.

### 6.3. Computational Cost Comparison

From a computational standpoint, the complete DBEA pipeline was also evaluated in terms of processing time. In our implementation, using vibration windows of $T_{\mathrm{env}}=1$ s (corresponding to 40,000 samples), the end-to-end processing time per window—including envelope computation, FFT, band energy calculation, and centroid-based classification—was approximately 2.93 ms. This corresponds to processing about 341 windows per second, i.e., a real-time factor of RTF≈0.0029, meaning that only 0.29% of the window duration is required for computation. The same order of processing time was observed when applying the DBEA pipeline to the CWRU rolling-bearing dataset, indicating that the method remains computationally light even on a widely used benchmark. These results indicate that the proposed non-neural, band energy-based approach is less demanding than typical deep learning architectures or advanced deconvolution schemes (e.g., DCN/ConvNeXt-based models [28] or CNN-LSTM-GRU networks trained on CWRU data [27]), while still providing the high diagnostic performance reported in Section 6.

## 7. Conclusions

Given the central role of three-phase induction motors (TIMs) in industrial applications, reliable techniques for the early detection of faults in their critical components remain essential. This work proposes a new vibration-based algorithm, the Discriminative Band Energy Analysis (DBEA), to classify strongly incipient faults occurring concurrently in bearings and rotors using low-cost vibration sensors while considering different steady-state operating conditions (load level and supply-voltage unbalance).

The experimental results showed a well-defined separation in the DBEA feature space for the investigated fault conditions and operating scenarios. The validation procedure based on a nearest-centroid classifier confirmed the robustness of the approach, yielding low misclassification rates (on the order of 4.2% for a single specific condition) for two orthogonal vibration directions. Importantly, the remaining confusions were concentrated mainly among very closely spaced bearing wear levels, which is consistent with the inherently small physical differences between these strongly incipient conditions and with the observed overlap in conventional baseline indicators. Overall, the results support the use of band energy features extracted from AM-demodulated vibration envelopes as a practical representation for simultaneous incipient bearing and rotor fault diagnosis.

The present study also has limitations. First, the evaluation was performed under distinct steady-state operating points (100% and 80% load, and a 5% supply-voltage unbalance), and the behavior under continuously time-varying speed/load was not assessed. Second, although the method is robust for the tested parameter ranges, its application depends on suitable choices of the envelope window length and the analysis bandwidth. Additional sensor configurations and mounting locations were not explored in this work.

Future work may address these points by performing a more systematic parameter optimization procedure, extending the experimental campaign to additional electrical and mechanical fault types and sensor placements, and implementing and benchmarking the DBEA pipeline on embedded/portable hardware for real-time condition monitoring. Such extensions will further assess the generality of the proposed approach and its suitability for compact, low-cost industrial diagnostic systems.

## Figures and Tables

**Table 1:** Rated parameters of the induction motor and DC generator.

Parameter	Induction Motor	DC Generator
Rated power	5 hp	10 kW
Rated line voltage	220/380 V	380 V (armature)
Rated current	14.1 A	26.6 A (armature)
Number of poles	4	–
Field voltage	–	440 V
Field current	–	1.5 A
Bearing	SKF 6205	–
Nominal Speed	1740 RPM	–

**Table 2:** Test combinations for bearing fault level, rotor condition, and load/voltage scenarios (30 conditions, 80 repetitions each).

ID	Fault [μm]	Rotor Condition	Load	Voltage Condition	Repetitions
1	—	Healthy	100%	100% in all three phases	80
2	—	Healthy	80%	100% in all three phases	80
3	—	Healthy	100%	5% decrease in one phase	80
4	—	Failed	100%	100% in all three phases	80
5	—	Failed	80%	100% in all three phases	80
6	—	Failed	100%	5% decrease in one phase	80
7	100–130	Healthy	100%	100% in all three phases	80
8	100–130	Healthy	80%	100% in all three phases	80
9	100–130	Healthy	100%	5% decrease in one phase	80
10	100–130	Failed	100%	100% in all three phases	80
11	100–130	Failed	80%	100% in all three phases	80
12	100–130	Failed	100%	5% decrease in one phase	80
13	150–180	Healthy	100%	100% in all three phases	80
14	150–180	Healthy	80%	100% in all three phases	80
15	150–180	Healthy	100%	5% decrease in one phase	80
16	150–180	Failed	100%	100% in all three phases	80
17	150–180	Failed	80%	100% in all three phases	80
18	150–180	Failed	100%	5% decrease in one phase	80
19	200–250	Healthy	100%	100% in all three phases	80
20	200–250	Healthy	80%	100% in all three phases	80
21	200–250	Healthy	100%	5% decrease in one phase	80
22	200–250	Failed	100%	100% in all three phases	80
23	200–250	Failed	80%	100% in all three phases	80
24	200–250	Failed	100%	5% decrease in one phase	80
25	450–480	Healthy	100%	100% in all three phases	80
26	450–480	Healthy	80%	100% in all three phases	80
27	450–480	Healthy	100%	5% decrease in one phase	80
28	450–480	Failed	100%	100% in all three phases	80
29	450–480	Failed	80%	100% in all three phases	80
30	450–480	Failed	100%	5% decrease in one phase	80
**Total**					**2400**

**Table 3:** Normalized average DBEA results for different parameters (normalization by the maximum value across all listed cases).

Sensor	Envelope Window Length	Bandwidth	Normalized Average DBEA
S2	100	50 Hz	1.00
S1	100	50 Hz	0.99
S1	100	20 Hz	0.25
S2	100	20 Hz	0.25
S2	200	50 Hz	0.52
S1	200	50 Hz	0.46
S1	200	20 Hz	0.26
S2	200	20 Hz	0.26
S2	500	50 Hz	0.55
S1	500	50 Hz	0.50
S1	500	20 Hz	0.26
S2	500	20 Hz	0.26
S2	1000	50 Hz	0.70
S1	1000	50 Hz	0.66
S1	1000	20 Hz	0.27
S2	1000	20 Hz	0.26
S2	5000	50 Hz	0.86
S1	5000	50 Hz	0.81
S1	5000	20 Hz	0.22
S2	5000	20 Hz	0.20

**Table 4:** Per-class comparison of Precision (P), Recall (R), and F1-score.

DBEA Overall Accuracy: 0.93 ± 0.024					Envelope Overall Accuracy: 0.47 ± 0.042				
Class	Acc	P	R	F1	Class	Acc	P	R	F1
1	0.99 ± 0.007	0.93 ± 0.058	1.00 ± 0.000	0.96 ± 0.031	1	0.93 ± 0.021	0.65 ± 0.121	0.61 ± 0.134	0.62 ± 0.114
2	0.99 ± 0.011	0.98 ± 0.034	0.94 ± 0.111	0.96 ± 0.066	2	0.87 ± 0.023	0.40 ± 0.087	0.54 ± 0.142	0.45 ± 0.099
3	0.99 ± 0.011	0.95 ± 0.086	0.98 ± 0.035	0.97 ± 0.051	3	0.86 ± 0.028	0.34 ± 0.110	0.39 ± 0.134	0.36 ± 0.115
4	1.00 ± 0.000	1.00 ± 0.000	1.00 ± 0.000	1.00 ± 0.000	4	0.89 ± 0.023	0.43 ± 0.127	0.41 ± 0.169	0.41 ± 0.141
5	0.99 ± 0.007	1.00 ± 0.000	0.92 ± 0.068	0.96 ± 0.037	5	0.89 ± 0.021	0.44 ± 0.171	0.29 ± 0.125	0.34 ± 0.136
6	0.95 ± 0.020	0.75 ± 0.110	0.71 ± 0.126	0.72 ± 0.115	6	0.92 ± 0.021	0.58 ± 0.088	0.76 ± 0.124	0.66 ± 0.087
7	0.95 ± 0.020	0.72 ± 0.096	0.76 ± 0.114	0.74 ± 0.100	7	0.88 ± 0.017	0.21 ± 0.242	0.07 ± 0.062	0.10 ± 0.093
8	1.00 ± 0.000	1.00 ± 0.000	1.00 ± 0.000	1.00 ± 0.000	8	0.87 ± 0.025	0.37 ± 0.114	0.38 ± 0.130	0.36 ± 0.109
9	1.00 ± 0.000	1.00 ± 0.000	1.00 ± 0.000	1.00 ± 0.000	9	0.96 ± 0.015	0.83 ± 0.098	0.74 ± 0.113	0.78 ± 0.080
10	1.00 ± 0.000	1.00 ± 0.000	1.00 ± 0.000	1.00 ± 0.000	10	0.87 ± 0.025	0.39 ± 0.093	0.52 ± 0.150	0.44 ± 0.105
**Kurtosis** **Overall Accuracy: 0.40 ± 0.028**					**RMS** **Overall Accuracy: 0.61 ± 0.032**				
**Class**	**Acc**	**P**	**R**	**F1**	**Class**	**Acc**	**P**	**R**	**F1**
1	0.87 ± 0.018	0.25 ± 0.156	0.15 ± 0.099	0.19 ± 0.111	1	0.97 ± 0.014	0.82 ± 0.080	0.92 ± 0.079	0.86 ± 0.063
2	0.87 ± 0.039	0.09 ± 0.155	0.14 ± 0.236	0.10 ± 0.160	2	0.89 ± 0.022	0.48 ± 0.078	0.66 ± 0.121	0.55 ± 0.084
3	0.94 ± 0.020	0.66 ± 0.095	0.81 ± 0.122	0.72 ± 0.087	3	0.92 ± 0.018	0.60 ± 0.092	0.64 ± 0.118	0.61 ± 0.087
4	0.98 ± 0.012	0.85 ± 0.076	0.93 ± 0.065	0.88 ± 0.055	4	1.00 ± 0.000	1.00 ± 0.000	1.00 ± 0.000	1.00 ± 0.000
5	0.82 ± 0.025	0.12 ± 0.084	0.12 ± 0.089	0.12 ± 0.083	5	0.92 ± 0.018	0.68 ± 0.157	0.45 ± 0.127	0.54 ± 0.123
6	0.80 ± 0.030	0.27 ± 0.056	0.58 ± 0.138	0.37 ± 0.075	6	0.89 ± 0.023	0.46 ± 0.115	0.47 ± 0.132	0.46 ± 0.110
7	0.87 ± 0.019	0.22 ± 0.160	0.12 ± 0.093	0.15 ± 0.107	7	0.84 ± 0.024	0.16 ± 0.095	0.15 ± 0.096	0.15 ± 0.092
8	0.88 ± 0.018	0.25 ± 0.189	0.13 ± 0.096	0.17 ± 0.115	8	0.85 ± 0.024	0.22 ± 0.119	0.18 ± 0.102	0.19 ± 0.106
9	0.98 ± 0.012	0.95 ± 0.056	0.83 ± 0.100	0.88 ± 0.069	9	1.00 ± 0.000	1.00 ± 0.000	1.00 ± 0.000	1.00 ± 0.000
10	0.81 ± 0.057	0.14 ± 0.121	0.23 ± 0.161	0.16 ± 0.107	10	0.94 ± 0.018	0.72 ± 0.122	0.67 ± 0.137	0.68 ± 0.098

**Table 5:** Per-class metrics using repeated stratified 10-fold cross-validation in CWRU dataset.

Class	Accuracy	Precision	Recall	F1-Score
1	1.00 ± 0.000	1.00 ± 0.000	1.00 ± 0.000	1.00 ± 0.000
2	0.97 ± 0.019	0.97 ± 0.047	0.87 ± 0.089	0.91 ± 0.055
3	0.97 ± 0.019	0.89 ± 0.070	0.97 ± 0.047	0.93 ± 0.044
4	1.00 ± 0.005	1.00 ± 0.000	0.99 ± 0.023	1.00 ± 0.012
5	1.00 ± 0.005	0.99 ± 0.021	1.00 ± 0.000	1.00 ± 0.011
**Average**	0.99	0.97	0.97	0.97

## Data Availability

The original contributions presented in this study are included in the article. Further inquiries can be directed to the corresponding author upon reasonable request.
